# Longer Sleep – Slimmer Kids: The ENERGY-Project

**DOI:** 10.1371/journal.pone.0059522

**Published:** 2013-03-19

**Authors:** Teatske M. Altenburg, Mai J. M. Chinapaw, Elise T. W. van der Knaap, Johannes Brug, Yannis Manios, Amika S. Singh

**Affiliations:** 1 Department of Public and Occupational Health, VU University Medical Center, EMGO Institute for Health and Care Research, Amsterdam, The Netherlands; 2 Department of Epidemiology and Biostatistics, EMGO Institute for Health and Care Research, VU University Medical Center, Amsterdam, The Netherlands; 3 Department of Nutrition and Dietetics, Harokopio University, Athens, Greece; University College London, United Kingdom

## Abstract

**Background:**

Few studies have differentiated between weekday and weekend day sleep duration in their association with indicators of weight status in children. Therefore, we examined the association of week and weekend day sleep duration with indicators of body composition in 10–12 year old European school children.

**Methods and Findings:**

Multi-level linear regression analysis was performed to examine the association between parent-reported week and weekend day sleep duration and objectively assessed child BMI and WC, adjusting for socio-demographic variables and energy balanced related behaviours EBRBs (i.e. dietary, physical and sedentary behaviour). Compared to sleeping 10 hrs/night or more, sleeping on average less than 10 hrs/night during weekdays was associated with higher BMI (for example, B = 0.86 and CI = [0.27;1.45] when sleeping ≤7 hrs) and WC (for example, B = 1.99 and CI = [0.32;3.65] when sleeping ≤7 hrs). Sleeping 9 hrs/night during weekend days, but not ≤8 hrs, was associated with higher WC (B = 0.66; CI = [0.04;1.28]) compared to sleeping more than 10 hrs/night. Average (week and weekend) sleep duration less than 10 hrs/night was associated with higher values for BMI (B = 0.98; CI = [0.24;1.73] and WC (B = 2.35; CI = [0.08;4.31]).

**Conclusions:**

Weekday sleep duration seems more strongly associated with body composition in European school children than weekend day sleep duration. Promoting adequate sleep duration may contribute to healthy weight in children.

## Introduction

The prevalence of overweight and obesity among children in Europe has increased over the last decades, with vast differences between individual countries [1]. Obesity in childhood is linked to several serious health implications [2] and childhood overweight is a strong predictor of being overweight in adulthood [3]. Coinciding with the increase in childhood overweight and obesity, recent reports have shown that the sleep duration of children aged 10–15 years has declined with on average half an hour over the past few decades [4]. Results of a recent review demonstrate that there is increasing evidence demonstrating that short sleep duration is associated with indicators of weight status in children and adolescents, based on findings from both cross-sectional and longitudinal studies. However, due to methodological limitations such as study design and consistency of covariates, it was concluded that it still remains inconclusive whether shortened sleep is a risk factor for overweight and obesity [5].

One possible mechanism of this association is that people who sleep for short durations have reduced serum levels of leptin and elevated levels of ghrelin, contributing to the development of overweight [6]. In addition, people with short sleep durations have decreased levels of growth hormone [7], resulting in diminished lipolysis in adipose tissue [8]. Other possible mechanisms include an increased timeframe to eat and decreased physical activity due to fatigue and daytime sleepiness in people with short sleep duration [9].

Several studies have reported differences between children’s weekday and weekend day sleep duration, with shorter durations on weekdays compared to weekend days [10]–[12]. To the best of our knowledge, only two of these studies have differentiated between weekday and weekend day sleep duration in their reports on association with indicators of weight status in children, showing conflicting results [10], [12]. A possible explanation for these conflicting results could be a lack of consistency of covariates included in the analyses. For example, only one of these studies adjusted for energy balance related behaviours (EBRBs), i.e. dietary, physical activity and sedentary behaviours [10]. Since previous studies showed that EBRBs are associated with both sleep and overweight [12]–[14], it is important to consider these variables as covariates in studies further exploring the association between sleep and weight status.

In addition to adjusting for EBRBs, in none of the studies examining the separate association of weekday and weekend day sleep duration and weight status, adjustments were made for weekend day sleep duration in the association of weekday sleep duration and weight status, vice versa. This adjustment is important since compensation for short weekday sleep duration by sleeping longer during weekend days is associated with decreased risk of childhood overweight and obesity [12].

The EuropeaN Energy balance Research to prevent excessive weight Gain among Youth (ENERGY)-project aims to establish a school- and family-involved intervention program, which advances and supports healthy EBRBS across Europe [15]. The present study uses data of the cross-sectional study of the ENERGY-project. The broad spectrum of these data enables us to assess sleep duration separately for weekdays and weekend days. In addition, we were able to consider socio-demographics variables and EBRBs, which may potentially confound the association between sleep duration and weight status. The aim of the current study is to examine the association between weekday (independent of weekend day), weekend day (independent of weekday) and average sleep duration and indicators of weight status (i.e. body mass index (BMI) and waist circumference (WC)) in schoolchildren across Europe.

## Methods

### Ethics Statement

This study was approved by the ethics committee of the VU University Medical Center and is in accordance with the Declaration of Helsinki. All participants and their parents provided written informed consent prior to their inclusion in the study.

### Study Design and Participants

The study sample consisted of 10–12 year old children and their parents who were enrolled in the ENERGY cross-sectional school-based survey, as part of the ENERGY-project [16]. Data of 7234 children were collected between March and July 2010 in seven European countries: Belgium, Greece, Hungary, The Netherlands, Norway, Slovenia and Spain. Questionnaires used in this survey were pilot-tested and evaluated for test-retest reliability and construct validity [17], [18]. Children with missing data on anthropometrics and sleep duration were excluded from the present analysis, resulting in a final sample size of 5757 children (46% boys). Of this sample, complete data for parent-reported weekday and weekend day sleep duration were available for 5733 and 5540 children respectively.

All participating countries obtained ethical clearance from the relevant ethical committees and ministries. Following the school’s agreement, parents received a letter explaining the study purpose and were asked for written informed consent for their child’s and own participation.

### Sleep Duration

Sleep duration (hrs/night) was parent reported, using the question ‘*How many hours of sleep does your child usually have during the night?*’. The response options with six possible answers ranged from ‘6 hours or less/per night’ to ‘More than 10 hours/per night’. The question was asked separately for weekdays and weekend days, showing good test-retest reliability/construct validity (ICC = 0.81/0.80 and 0.80/0.79).

### Indicators of Weight Status

Indicators of weight status included BMI and WC. Body weight (kg), height (cm), and WC were measured in light clothing and without shoes, using a standardised protocol. Weight was measured to the nearest 0.1 kg using a calibrated electronic scale (SECA 861). Height was measured to the nearest 0.1 cm using a Seca Leicester Portable stadiometer and WC was measured to the nearest 0.1 cm with the circumference measuring band (SECA 201). Two readings of each measurement were obtained. If these two readings differed more than 1%, a third measure was taken. BMI was calculated as weight (kg) divided by height squared (m^2^) and was used as an indicator of overweight and obesity. Childhood overweight and obesity were defined according to the Cole criteria [19].

### Covariates

Analyses were adjusted for socio-demographic variables and EBRBs. Socio-demographic variables included age, gender, parental education and parental ethnicity. Parental education was self-reported by the parents, and categorized as being high (i.e. at least one parent more than 14 years of education) or low (i.e. both parents less than 14 years of education). Parental ethnicity was assessed by parent-report and categorized as one biological parent born in the country of administration or not.

EBRBs included frequency of taking breakfast, sugared drink consumption, physical activity and screen time. Frequency of taking breakfast was assessed by asking the children how many days per week they normally had breakfast, with response options ranging from zero to 7 days per week. Sugared drink consumption was assessed using two food frequency questions. Children were asked how often (number of days per week) and how much (serving size) they normally drink soft drinks and fruit juices. Mean intake (ml) of sugared drink consumption was calculated by multiplying the number of days per week and the amount per day divided by 7. Physical activity was assessed by asking how much time (minutes) per week (e.g. number of days per week and how many minutes/hours) the children were physically active. Separate questions were asked for sports and active transport (going to school by bike or by foot). Screen time was assessed by asking the children how much time (minutes) per day they spent watching television and using the computer.

### Statistics

Descriptive subject characteristics were given as mean values (± SD) and percentages. Gender differences were examined using chi-square tests for categorical data and Student’s unpaired *t*-tests for continuous data (SPSS for Windows, version 18.0; Chicago, IL, USA).

To examine the association between sleep duration and BMI and WC, weekday and weekend day sleep duration was categorised as sleeping ≤7, 8, 9 and ≥10 hours per night), with sleeping ≥10 hours per night as the reference category. Weighed average sleep duration was calculated using the following equation: average sleep = (5 × weekday sleep duration +2 × weekend day sleep duration)/7. Multi-level linear regression analysis (MLwiN, version 2.2; Center for Multilevel Modeling, University of Bristol, England) with a three-level structure (country, school, and child) was used (model 1). This technique enables the adjustment of the regression coefficients for the clustering of observations within countries, schools and/or classes. Separate analyses were run for weekday, weekend day and average sleep duration. Adjustments were made for socio-demographics (i.e. age, gender, parental education, parental ethnicity; model 1) and EBRBs (i.e. breakfast frequency, sugared drink consumption, physical activity and screen time; model 2). In addition, a third model was run including weekend day sleep duration as covariate in the association with weekday sleep duration, and weekday sleep duration in the association with weekend day sleep duration. This model was not run when average sleep duration was modelled as the predictor variable.

## Results

### Participant Characteristics

Participant characteristics are presented in Table 1. Boys had a significantly larger WC than girls, and significantly fewer boys were overweight and obese. Sleep duration during weekend days was significantly shorter for boys than for girls.

**Table 1:** Study participant characteristics (mean (SD)).

	All (N = 5757)	Boys (N = 2662)	Girls (N = 3095)
Age (years)	11.6	11.6 (0.7)	11.6 (0.7)
Weight (kg)	44.3	44.4 (10.5)	44.3 (10.3)
Height (cm)	151.7	151.5 (8.5)	151.8 (8.3)
WC (cm)a, c	66.4	67.5 (9.3)	65.5 (8.5)
BMI (kg/m^2^)	19.1	19.2 (3.3)	19.0 (3.3)
Overweight (%)b, c	19.5	21.4	17.8
Obesity (%)^d^	4.7	5.3	4.2
Sleep duration			
Weekdays (%)^a^			
≤7 h	3.3	3.7	3.0
8	20.8	19.9	21.6
9	46.1	46.7	45.6
≥10	29.7	29.6	29.8
Weekend days (%)a, c			
≤7 hr	2.5	2.7	2.3
8	10.3	12.0	8.8
9	31.0	34.3	28.1
≥10	56.2	51.0	60.8
Average (%)^d^			
≤7 hr	1.9	2.3	1.6
8	14.7	15.7	13.9
9	46.3	48.6	49.9
≥10	34.1	33.4	34.6

a Divergent number of participants:

WC: N = 2658 for boys and N = 3088 for girls; Weekday sleep duration: N = 2654 for boys and N = 3079 for girls; Weekend day sleep duration: N = 2574 for boys and N = 2966 for girls; Average sleep duration: N = 2566 for boys and 2950 for girls.

b Overweight only.

c *P*≤0.001 for sex difference.

d *P*≤0.05 for sex difference.

### Sleep Duration in European Countries

Parent-reported child sleep duration of 9 hours per night was most common (Table 2). However, parents of children in Greece were more likely to report 8 hours of sleep per night, whereas for Belgian and Dutch children 10 hours sleep per night was most often reported. During weekend days the majority of the parents reported child sleep duration of 10 hours or more across all countries.

**Table 2:** Prevalence rates of sleep duration (%) across European countries.

	Belgium	Greece	Hungary	Netherlands	Norway	Slovenia	Spain
Weekdays	(N = 737)	(N = 983)	(N = 915)	(N = 375)	(N = 813)	(N = 974)	(N = 936)
≤7 hr	0.9	10.1	3.6	1.3	0.9	3.0	1.3
8	7.1	43.3	28.0	7.5	14.9	18.0	13.5
9	30.8	35.2	52.2	29.1	58.3	53.7	51.7
≥10	61.2	11.3	16.0	62.1	26.0	24.4	33.5
Weekend days	(N = 707)	(N = 974)	(N = 890)	(N = 349)	(N = 776)	(N = 932)	(N = 912)
≤7 hr	0.4	6.3	2.5	1.7	1.3	3.0	1.0
8	9.1	13.1	8.5	13.2	10.8	11.6	6.9
9	27.3	29.3	29.3	33.2	37.0	35.3	26.9
≥10	63.2	51.3	59.7	51.9	50.9	50.1	65.2
Average	(N = 700)	(N = 974)	(N = 888)	(N = 216)	(N = 775)	(N = 928)	(N = 904)
≤7 hr	0.7	5.2	2.5	1.2	0.3	1.7	0.8
8	5.1	30.3	17.6	6.6	11.0	14.2	9.5
9	31.3	48.3	56.4	30.0	57.9	56.3	50.0
≥10	62.9	16.2	23.5	62.2	30.8	27.8	39.7

### Association between Weekday Sleep Duration and Indicators of Weight Status

The associations between sleep duration during weekdays and indicators of weight status are presented in Table 3. Compared to sleeping 10 hours or more, sleeping on average less than 10 hours per night during weekdays was associated with higher values of BMI and WC, independent of socio-demographic variables, EBRBs and weekend day sleep duration (model 3). When sleeping for example 7 hours or less instead of 10 hours per night during weekdays, BMI was on average 0.86 kg/m^2^ higher and WC was on average 1.99 cm higher.

**Table 3:** Cross-sectional associations (regression coefficients (b) and 95% confidence intervals (95% CI)) between weekday, weekend day and average sleep duration and BMI and WC in schoolchildren across Europe.

	(A) Week day		(B) Weekend day		(C) Average	
Sleep duration (hr)	BMI	WC	BMI	WC	BMI	WC
**Model 1**						
10	1.0	1.0	1.0	1.0	1.0	1.0
9	0.37 [0.16; 0.59]*	0.93 [0.27; 1.58]*	0.25 [0.03; 0.49]*	0.89 [0.30; 1.47]*	0.41 [0.18; 0.65]*	0.96 [0.45; 1.71]*
8	0.37 [0.15; 0.59]*	1.76 [0.95; 2.57]*	0.20 [−0.13; 0.65]	0.78 [−0.08; 1.65]	0.61 [0.28; 0.93]*	1.31 [1.02; 2.09]*
≤7	0.89 [0.36; 1.41]*	1.90 [0.42; 3.37]*	0.29 [−0.34; 1.10]	0.64 [−1.00; 2.28]	0.92 [0.21; 1.64]*	2.37 [1.02; 4.31]*
**Model 2**						
10	1.0	1.0	1.0	1.0	1.0	1.0
9	0.37 [0.15; 0.59]*	0.98 [0.30; 1.66]*	0.30 [0.06; 0.53]*	0.89 [0.28; 1.50]*	0.44 [0.19; 0.69]*	1.06 [0.23; 1.71]*
8	0.68 [0.39; 0.96]*	1.56 [0.72; 2.39]*	0.15 [−0.19; 0.50]	0.59 [−0.30; 1.49]	0.64 [0.31; 0.98]*	1.20 [0.17; 2.09]*
≤7	0.90 [0.35; 1.45]*	1.87 [0.33; 3.41]*	0.43 [−0.22; 1.08]	0.87 [−0.84; 2.59]	0.98 [0.24; 1.73]*	2.35 [0.08; 4.31]*
**Model 3**						
10	1.0	1.0	1.0	1.0	N/A	N/A
9	0.31 [0.081; 0.54]*	0.88 [0.17; 1.58]*	0.18 [−0.06; 0.42]	0.66 [0.04; 1.28]*	N/A	N/A
8	0.66 [0.36; 0.97]*	1.55 [0.66; 2.44]*	−0.06 [−0.41; 0.30]	0.17 [−0.76; 1.09]	N/A	N/A
≤7	0.86 [0.27; 1.45]*	1.99 [0.32; 3.65]*	0.14 [−0.54; 0.82]	0.27 [−1.52; 2.05]	N/A	N/A

Model 1: Adjusted for age, gender, parental BMI, parental education, parental employment, biological background of the parents, family composition, home-situation, television in bedroom and set daily routine for bedtime;

Model 2: Additionally adjusted for frequency of taking breakfast, sugared drink consumption, physical activity and screen time;

Model 3: Additionally adjusted for weekend (A) or week (B) sleep duration.

Note that model 3 was not run for average sleep duration, since it comprises both weekday and weekend day sleep duration.

* = Significant association between weekday or weekend day sleep duration (hr) and BMI/WC.

### Association between Weekend Day Sleep Duration and Indicators of Weight Status

The associations between sleep duration during weekend days and indicators of weight status are presented in Table 3. Compared to sleeping 10 hours or more, sleeping 9 hours (but not 8 hours or less), during weekend days was associated with higher values of WC (on average 0.66 cm higher), but not BMI, independent of socio-demographic variables, EBRBs and weekday sleep duration (model 3).

### Association between Average Sleep Duration and Indicators of Weight Status

The associations between average sleep duration and indicators of weight status are presented in Table 3. Compared to sleeping 10 hours or more, sleeping on average less than 10 hours per night was associated with higher values for BMI and WC, independent of socio-demographic variables and EBRBs (model 3). When sleeping for example 7 hours or less instead of 10 hours per night, mean BMI was 0.98 kg/m^2^ higher and mean WC was 2.35 cm higher.

## Discussion

The aim of this study was to examine the association between weekday, weekend day and average sleep duration and BMI and WC in almost 6000 schoolchildren across seven European countries. Sleep duration was associated with indicators of weight status in European 10–12 year old children, independent of weekend day sleep duration, several EBRBs, demographic and socio-environmental variables. This association was stronger for weekdays than for weekend days.

Our results support previous findings from cross-sectional [20]–[22] and longitudinal studies [19], [23], [24] in comparable age group reporting significant inverse associations between average sleep duration and indicators of weight status in children. In addition, our findings of an inverse association between weekday sleep duration and indicators of weight status in children are in line with previous studies [10], [12], although Lytle et al. [10] reported that this association was not consistent in girls. In contrast to the study of Wing [12], we could not confirm an inverse association between weekend day sleep duration and weight status in children. One possible explanation could be that in our study adjustments were made for several EBRBs, demographic and socio-environmental variables, whereas in the study of Wing et al [12], adjustments were limited to age, gender, watching TV, time on homework, parental education and eating 1 hour before going to bed.

Although we demonstrated inverse associations between both average and weekday sleep duration and indicators of weight status, regression coefficients were smaller after adjustment for weekend day sleep duration. In line with previous studies [12], [25], our results indicate that children might compensate their weekday sleep shortcoming during weekend days, thereby weakening the inverse association between weekday sleep duration and indicators of weight status. This emphasizes the importance of discriminating between weekday and weekend day sleep duration in the association with indicators of weight status in children.

We found a stronger association of sleep duration with indicators of weight status for weekdays compared to weekend days. One explanation might be that weekday sleep duration covers 5 days of the week, and weekend sleep duration only 2 days. Weekday sleep duration might therefore influence weight status to a larger extent than weekend day sleep duration. Another explanation might be that weekday sleep duration is easier to recall, since bedtimes during weekdays are more likely to adhere to a time schedule compared to weekend days. The more reliable reporting of weekday sleep duration might have strengthened the association with weight status.

The observed variation in weekday sleep duration between countries within Europe are in line with the findings of Hense et al [26]. The shorter sleep duration of children from southern European countries (e.g. Greece) compared to children from northern countries (e.g. Belgium) might be explained by less concerns of Greek parents about their children’s sleep habits, leading to more unstructured and flexible bedtimes [27]. It has also been observed that dinner times are much later at night in many southern European countries which might in turn lead to later bedtimes and thus shorter sleep duration of southern European children compared to northern European children [27].

Although the associations observed in the present study were independent of weekend day sleep duration, EBRBs, demographic and socio-environmental influences, it is possible that other variables that were not measured, such as parenting practices, parenting styles, having a TV in the bedroom and depression, may have confounded the associations found [5], [28], [29]. However, research reported by Landhuis et al [24] and Lumeng et al [19] showed that the association between short sleep duration and risk for childhood overweight was independent of parental style. Children with a TV in their bedroom have reported shorter sleep durations, possibly due to later bedtimes. Although data on television viewing at bedtime were not available, adjusting for having a television in the children’s bedroom did not change the association between sleep duration and indicators of weight status in the present study.

One limitation of the present study is the cross-sectional design, implicating that no conclusions about causality can be drawn. Second, sleep duration was parent reported and not objectively measured by polysomnography [30], and thus liable to social desirability bias. Third, sleep duration was assessed categorically with each category including a one hour sleep duration range, and no information on sleep latency and wake up time was questioned. Therefore, no information on sleep patterns was available. Unfortunately, we could not examine associations between sleep duration and indicators of weight status for each country separately, since the number of children in the shortest and longest sleep duration groups was relatively small per country. Finally, we did not measure pubertal stage, which could also confound or mediate the association between sleep duration and overweight, since pubertal stage is associated with overweight/obesity.

The study was part of the ENERGY-project, which provided a number of strengths. First, data on sleep duration on both weekdays and weekend days were obtained within the ENERGY-project, which allowed us to study both the association of weekday and weekend day sleep duration on indicators of weight status. Second, the present study includes a large representative sample of European children from different countries and different regions within each country. This large multinational sample allowed comparisons between the countries, concerning sleep duration, overweight and obesity. Third, a large variety of important EBRBs and demographic and socio-environmental variables were included in the analyses, which could potentially confound the association between sleep duration and overweight. Finally, data were collected according to a standardized protocol across the different countries and objectively measured anthropometrics were obtained.

### Conclusion

Our study results suggest that weekday sleep duration is more strongly associated with body composition in European school children than weekend day sleep duration, independent of EBRBs, demographic and socio-environmental influences. Promotion of longer sleep duration (e.g. at least 10 hours during weekdays) may therefore positively contribute to weight management in childhood.
